# The Chaperone and Redox Properties of CnoX Chaperedoxins Are Tailored to the Proteostatic Needs of Bacterial Species

**DOI:** 10.1128/mBio.01541-18

**Published:** 2018-11-27

**Authors:** Camille V. Goemans, François Beaufay, Isabelle S. Arts, Rym Agrebi, Didier Vertommen, Jean-François Collet

**Affiliations:** a Welbio, Brussels, Belgium; b de Duve Institute, Université catholique de Louvain, Brussels, Belgium; University of Michigan—Ann Arbor

**Keywords:** chaperones, oxidative stress, protein folding, thioredoxin

## Abstract

Hypochlorous acid (bleach), an oxidizing compound produced by neutrophils, turns the Escherichia coli chaperedoxin CnoX into a powerful holdase protecting its substrates from bleach-induced aggregation. CnoX is well conserved in bacteria, even in non-infectious species unlikely to encounter this oxidant, muddying the role of CnoX in these organisms. Here, we found that CnoX in the non-pathogenic aquatic bacterium Caulobacter crescentus functions as a holdase that efficiently protects 50 proteins from heat-induced aggregation. Remarkably, the chaperone activity of Caulobacter CnoX is constitutive. Like E. coli CnoX, Caulobacter CnoX transfers its substrates to DnaK/J/GrpE and GroEL/ES for refolding, indicating conservation of cooperation with GroEL/ES. Interestingly, Caulobacter CnoX exhibits thioredoxin oxidoreductase activity, by which it controls the redox state of 90 proteins. This function, which E. coli CnoX lacks, is likely welcome in a bacterium poorly equipped with antioxidant defenses. Thus, the redox and chaperone properties of CnoX chaperedoxins were fine-tuned during evolution to adapt these proteins to the specific needs of each species.

## Abstract (precis)

How proteins are protected from stress-induced aggregation is a crucial question in biology and a long-standing mystery. While a long series of landmark studies have provided important contributions to our current understanding of the proteostasis network, key fundamental questions remain unsolved. In this study, we show that the intrinsic features of the chaperedoxin CnoX, a folding factor that combines chaperone and redox protective function, have been tailored during evolution to fit to the specific needs of their host. Whereas Escherichia coli CnoX needs to be activated by bleach, a powerful oxidant produced by our immune system, its counterpart in Caulobacter crescentus, a bacterium living in bleach-free environments, is a constitutive chaperone. In addition, the redox properties of E. coli and C. crescentus CnoX also differ to best contribute to their respective cellular redox homeostasis. This work demonstrates how proteins from the same family have evolved to meet the needs of their hosts.

## INTRODUCTION

Like all living cells, bacteria express an intricate network of protein-folding factors that assist the folding of nascent polypeptides and protect their integrity despite ever-changing environmental conditions (1, 2). Maintaining protein homeostasis is a crucial and challenging task, especially because several stresses encountered by bacteria, including heat shock or oxidative stress, lead directly or indirectly to protein unfolding and aggregation (3, 4). When stress occurs and proteins unfold, holdase chaperones bind to unfolded polypeptides and prevent their often-irreversible aggregation. However, most holdases are unable to actively refold their substrate proteins after stress; their function consists of maintaining substrates in a folding-competent conformation and transferring them to foldases when conditions return to normal (5). The two major foldases in the bacterial cytoplasm are DnaK/J/GrpE and GroEL/ES, two molecular machineries that use cellular energy to promote the refolding of their substrates. DnaK, the bacterial ortholog of Hsp70, is a central foldase involved in the folding of newly synthesized polypeptides, the prevention of aggregation, and the refolding of misfolded and aggregated proteins (6). DnaK binds solvent-exposed hydrophobic regions of its substrates and promotes protein refolding in an ATP-driven process regulated by the co-chaperone DnaJ and the nucleotide exchange factor GrpE (6). Although most cytoplasmic proteins are thought to interact with DnaK/J/GrpE during folding (7), this system is not essential in Escherichia coli, in contrast to GroEL/ES. GroEL (Hsp60), along with its co-chaperone GroES (Hsp10), forms a cage-like multimeric structure with ATPase activity (8) that promotes the folding of ∼250 proteins in E. coli, including 67 that are essential (8, 9).

Under oxidative stress, cysteine and methionine residues can become oxidized, which can lead to protein unfolding and/or inactivation (10). Therefore, in addition to chaperones, bacteria are equipped with oxidoreductases that contribute to proteostasis by rescuing redox-sensitive residues from oxidation (11, 12). The major cytoplasmic oxidoreductases are the thioredoxins (Trxs), small antioxidant proteins that are conserved in all living organisms and catalyze the reduction of oxidized cysteine residues in proteins (13). Trxs share a structural fold that consists of five β-strands surrounded by four α-helices and display a strictly conserved WCGPC catalytic motif (13). After substrate reduction, the two cysteine residues of the catalytic site are oxidized to a disulfide. In order to remain active, Trxs are recycled by thioredoxin reductase (TrxR), a flavoenzyme that uses electrons derived from the cellular pool of NADPH to keep Trxs reduced (13). Note that many proteins with oxidoreductase activity possess a Trx fold and display a catalytic CXXC motif. The WCGPC motif is however typical of classical reducing Trx proteins (13).

Because of the link between oxidation and aggregation, protein protection requires well-orchestrated coordination between chaperones and redox catalysts. Recently, we reported the identification and characterization of E. coli CnoX (*Ec*CnoX [previously YbbN]), a key component of the hypochlorous acid (HOCl) protection network (14). HOCl, the active ingredient of bleach, is a powerful oxidant released by our immune system to kill bacteria, mostly by oxidizing their proteins and causing protein unfolding and aggregation (15, 16). *Ec*CnoX consists of two domains with complementary functions: an N-terminal Trx domain and a C-terminal tetratricopeptide repeat (TPR) domain (17). TPR motifs usually mediate protein-protein interactions and are common in folding factors (18). We previously determined that bleach turns *Ec*CnoX into a powerful holdase by causing the chlorination of several residues in the TPR domain (14). This modification renders the protein surface more hydrophobic, thereby increasing its affinity for unfolded polypeptide (14). After stress, *Ec*CnoX transfers its substrates to DnaK/J/E and GroEL/ES for refolding (14). Remarkably, *Ec*CnoX is the only holdase reported to date in any prokaryote or eukaryote that cooperates with the essential GroEL/ES machinery. In *Ec*CnoX, the N-terminal Trx domain, which displays an SXXC motif, does not exhibit oxidoreductase activity. However, this domain plays a key role during HOCl stress: a cysteine residue located away from the SXXC motif forms mixed-disulfide complexes with sensitive cysteines in CnoX substrates under stress, protecting them from irreversible overoxidation (14). Because *Ec*CnoX uniquely combines holdase and redox protective function, we called it a chaperedoxin (chaperone—redox) (14).

The dual function of *Ec*CnoX protects bacteria from the major HOCl-mediated damages, protein oxidation, and aggregation. Intriguingly, CnoX proteins are highly conserved among bacteria (see Fig. S1 in the supplemental material), including in species that are unlikely to encounter HOCl in their natural habitats. What is the function of CnoX in these organisms? To answer this question, we unraveled the function of CnoX from the alphaproteobacterium Caulobacter crescentus (*Cc*CnoX), a non-pathogenic aquatic bacterium that is a favorite model organism due to its asymmetric cell cycle. First, we found that *Cc*CnoX is a holdase that protects a wide range of substrate proteins from aggregation during thermal stress. Remarkably, the chaperone function of *Cc*CnoX is constitutive. Like *Ec*CnoX, *Cc*CnoX conserves the unique ability to transfer its substrates to both DnaK/J/GrpE and GroEL/ES for refolding. Finally, *Cc*CnoX exhibits classical thioredoxin oxidoreductase activity and protects 90 proteins from oxidation. Thus, although the features of *Cc*CnoX are strikingly distinct from those of *Ec*CnoX, *Cc*CnoX combines chaperone and redox functions; it is therefore also a chaperedoxin. We conclude that the structural and redox properties of chaperedoxins have been tailored during evolution to meet the needs of their host species.

10.1128/mBio.01541-18.7 FIG S1 Phylogenetic analysis of *Cc*CnoX. Download FIG S1, PDF file, 0.2 MB. Copyright © 2018 Goemans et al. 2018 Goemans et al. This content is distributed under the terms of the Creative Commons Attribution 4.0 International license.

## RESULTS

### *Cc*CnoX exhibits constitutive holdase activity.

Holdases are ATP-independent chaperones that protect their substrates from stress-induced aggregation by maintaining them in a folding-competent state (5). A widely used method to assess holdase function consists of measuring prevention of the aggregation of model substrates, such as citrate synthase (CS). CS aggregates when incubated at high temperatures—thermal aggregation—or when diluted into buffer after unfolding with guanidine hydrochloride—chemical aggregation (19, 20). This aggregation increases the light scattering of the solution and can be measured with a spectrophotometer (19, 20). We recently reported that while *Ec*CnoX does not exhibit chaperone activity, exposure to bleach converts it into a powerful holdase (*Ec*CnoX_HOCl_); *Ec*CnoX_HOCl_ prevents both thermally and chemically induced aggregation of CS, even at a 1:1 ratio (14). *Ec*CnoX_HOCl_ also suppressed the aggregation of luciferase (14), a second model substrate, indicating that it is active against structurally diverse substrates.

**FIG 1:**
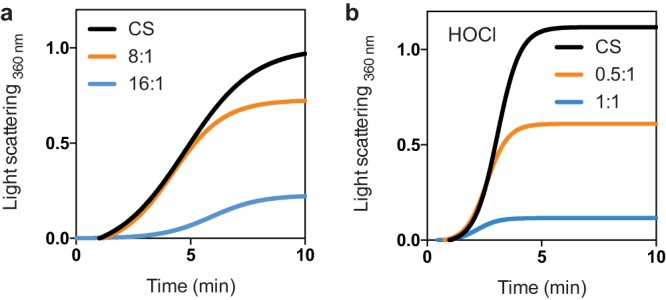
*Cc*CnoX is a constitutive holdase. (a) *Cc*CnoX inhibits the aggregation of thermally (43°C) denatured CS when present at high ratios. This test was performed in triplicate; this panel shows representative results. Additional results appear in the supplemental material (see Fig. S2A). (b) HOCl-treated *Cc*CnoX inhibits the aggregation of thermally (43°C) denatured CS. This test was performed in triplicate; this panel shows representative results. Additional results are presented in Fig. S2B.

*Cc*CnoX is expressed in a bacterium that is unlikely to encounter bleach in its natural environment—it is unlikely to be challenged by immune cells or by free-floating HOCl—thus raising the question of its function and potential activation mechanism. We hypothesized that *Cc*CnoX also displays holdase activity, but that this function does not need to be activated. To test this hypothesis, *Cc*CnoX was incubated with CS at 43°C, a condition that induces CS unfolding. Remarkably, *Cc*CnoX protected CS from thermal aggregation when present in sufficient amounts (from 8:1 *Cc*CnoX to CS) (Fig. 1a), whereas addition of *Ec*CnoX, as previously reported, had no impact (14). Thus, unlike its E. coli counterpart, *Cc*CnoX does not require prior activation to function as a holdase. Treatment of *Cc*CnoX with HOCl strongly increased its chaperone activity (Fig. 1b), but the physiological relevance of this result remains unclear.

10.1128/mBio.01541-18.8 FIG S2 Holdase experiment in triplicate. Download FIG S2, PDF file, 0.2 MB. Copyright © 2018 Goemans et al. 2018 Goemans et al. This content is distributed under the terms of the Creative Commons Attribution 4.0 International license.

### The surface of *Cc*CnoX is more hydrophobic than that of *Ec*CnoX.

**FIG 2:**
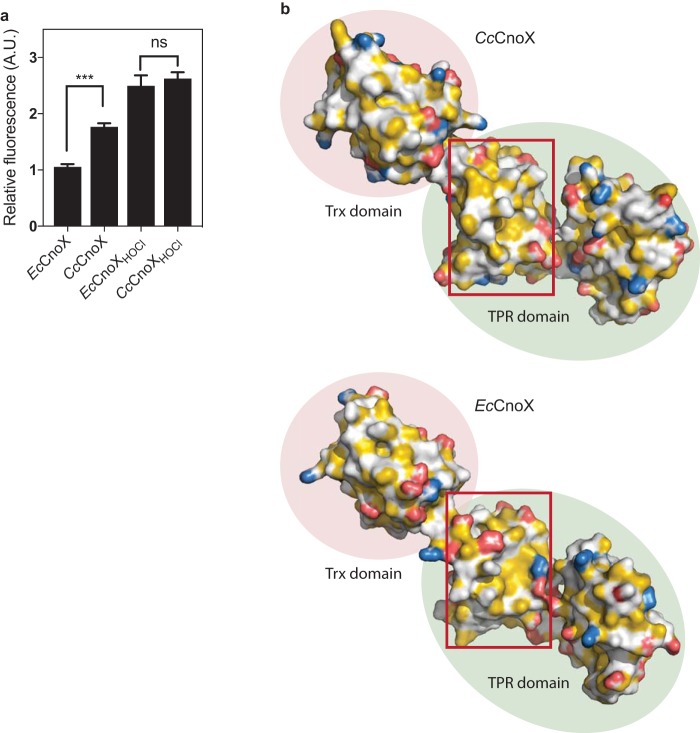
The surface of *Cc*CnoX is more hydrophobic than that of *Ec*CnoX. (a) The surface of *Cc*CnoX is more hydrophobic than that of *Ec*CnoX. After HOCl treatment, the hydrophobicities of both proteins are similar. Nile red was incubated with *Cc*CnoX and *Ec*CnoX (Materials and Methods), and fluorescence was measured in arbitrary units (A.U.) at 630 nm. This experiment was performed in triplicate; the fluorescence of *Ec*CnoX was set to 1. Error bars denote standard deviation. Differences were evaluated with Student's *t* test. ns, not significant (*P* > 0.05). ***, *P* < 0.001. (b) Comparison of the surfaces of *Cc*CnoX (model built with SWISS-MODEL) and *Ec*CnoX (structure from reference 17 [PDB no. 3QOU]) shows that the TPR domain of *Cc*CnoX displays larger hydrophobic patches than *Ec*CnoX (example in the red box). Hydrophobicity was detected and colored using YBR script (47). Hydrocarbon groups without polar substitutions are yellow, negatively charged oxygens of glutamate and aspartate are red, nitrogens of positively charged functional groups of lysine and arginine are blue, and all remaining atoms (including the polar backbone) are white (47).

We previously demonstrated that exposure of *Ec*CnoX to bleach causes the chlorination of surface-exposed residues, thus rendering the surface of this protein more hydrophobic and increasing its affinity for unfolded substrates (14). The observation that *Cc*CnoX exhibits holdase activity without bleach activation (Fig. 1a) suggested that its surface is intrinsically more hydrophobic than that of *Ec*CnoX. To test this hypothesis, both proteins were incubated with Nile red, an uncharged hydrophobic dye whose fluorescence increases with the hydrophobicity of the environment. Whereas after HOCl treatment, both proteins exhibited similar hydrophobicity levels, absolute fluorescence of the nontreated proteins was higher for C*c*CnoX than for *Ec*CnoX (Fig. 2a). We also built a structural model (Materials and Methods) for *Cc*CnoX, using SWISS-MODEL and *Ec*CnoX as a template (both proteins share 30% identity) (Fig. 2b). Comparison of the surfaces of hydrophobic patches revealed that *Cc*CnoX displays hydrophobic patches in its TPR domain that are larger (20% of its overall surface) than those of *Ec*CnoX (11% of its surface) (Fig. 2b). Taken together, these results indicate that the surface of *Cc*CnoX is substantially more hydrophobic than that of *Ec*CnoX, which likely explains why *Cc*CnoX has constitutive holdase activity.

### *Cc*CnoX cooperates with the GroEL and DnaK machineries.

Although holdases prevent protein aggregation by binding unfolded substrates, they are unable to actively help them to refold. This function is fulfilled by ATP-dependent foldases, which cooperate with holdases and, after stress, use cellular energy to help unfolded proteins reach their native state. The DnaK (Hsp70) and GroEL (Hsp60) machineries are two major foldase systems that are widely conserved in bacteria and eukaryotes (1, 2). We recently reported that *Ec*CnoX cooperates not only with the DnaK machinery (DnaK/J/GrpE) for refolding, like most studied holdases, but also with the GroEL system (GroEL/ES), which was unprecedented (14). This conclusion prompted us to investigate whether this property was conserved among CnoX homologs.

**FIG 3:**
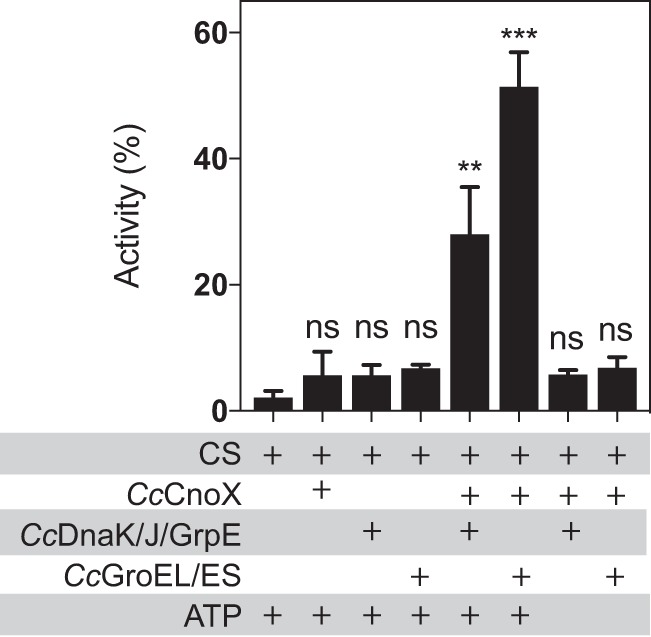
*Cc*CnoX cooperates with DnaK/J/GrpE and with GroEL/ES in C. crescentus. CS, chemically unfolded with guanidine hydrochloride, was diluted in refolding buffer containing various combinations of *Cc*CnoX (6:1 ratio to CS) with DnaK/J/GprE, GroEL/ES, and ATP (see Materials and Methods). The recovered activity of CS serves as a proxy to quantify CS refolding. *Cc*CnoX transfers CS to both *Cc*GroEL/ES and *Cc*DnaK/J/GrpE systems for refolding. Mean values from three independent experiments are shown; error bars denote standard deviation. Differences were evaluated with Student's *t* test. ns, not significant (*P* > 0.05). **, *P* < 0.01; ***, *P* < 0.001.

To that end, we investigated the ability of *Cc*CnoX to cooperate with the DnaK/J/GrpE and GroEL/ES systems of C. crescentus, using well-established CS refolding assays (20). Briefly, chemically denatured CS was first diluted into buffer with or without *Cc*CnoX. Then, folding chaperones (DnaK/J/GrpE or GroEL/ES, purified from C. crescentus) and ATP were added and CS activity was measured. In the absence of any chaperone or in the presence of *Cc*CnoX alone, almost no CS activity was recovered (Fig. 3), confirming that *Cc*CnoX does not exhibit refolding activity. In contrast, addition of GroEL/ES or DnaK/J/GrpE led to ∼60% or ∼30% recovery of initial CS activity, respectively (Fig. 3). Thus, like *Ec*CnoX, *Cc*CnoX cooperates with both refolding systems, indicating that the ability of CnoX to function with both GroEL/ES and DnaK/J/GrpE is probably a general feature of CnoX proteins.

### *Cc*CnoX is an important player in the proteostasis network.

After establishing that *Cc*CnoX is a constitutive holdase (Fig. 1) that cooperates with GroEL/ES and DnaK/J/GrpE (Fig. 3), we sought to determine the specific role played by this protein in the proteostasis network of C. crescentus. In contrast to E. coli, whose protein folding network has been extensively studied, not much is known about how C. crescentus ensures and monitors the folding of its polypeptides. Both the DnaK and GroEL machineries are essential in C. crescentus (21–23) and, like in E. coli, their expression is under the control of RpoH (24), the alternative σ factor that controls the heat shock response. In the absence of stress, the expression of both DnaK and GroEL is cell cycle regulated (21–23). However, the physiological role of this regulation is poorly characterized.

**FIG 4:**
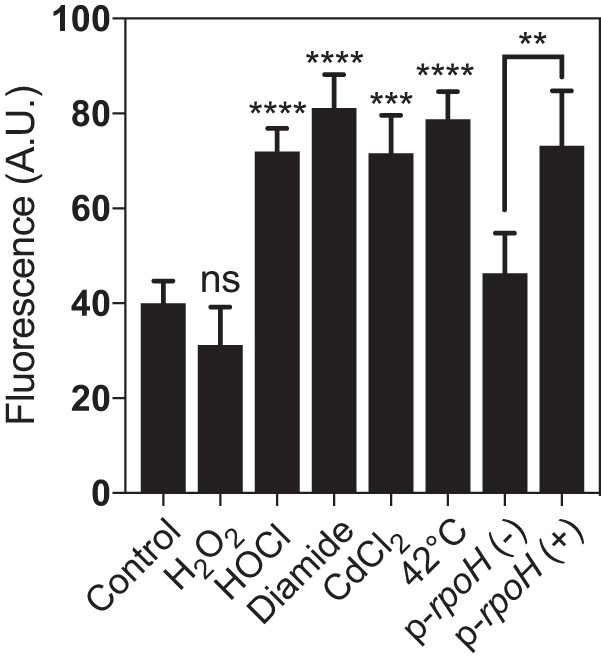
*Cc*CnoX belongs to the RpoH regulon. Relative fluorescence of the *CccnoX*::*CccnoX-gfp* strain was measured in triplicate (error bars denote standard deviation) under the indicated conditions. The last two bars on the right side of the graph show the fluorescence of a *CccnoX*::*CccnoX-gfp* strain harboring *rpoH* on a high-copy-number plasmid, in the absence (−) or the presence (+) of inducer. Differences were evaluated with Student's *t* test. ns, not significant (*P* > 0.05). **, *P* < 0.01; ***, *P* < 0.001; ****, *P* < 0.0001.

To test whether *Cc*CnoX belongs to the set of cellular defenses against protein misfolding and aggregation in C. crescentus, we monitored *Cc*CnoX production under conditions known to cause protein-folding stress. We replaced the chromosomal *CccnoX* gene with a *CccnoX-gfp* fusion inserted downstream of its native promoter (Materials and Methods). Addition of various compounds known to cause protein unfolding, including Cd^2+^, HOCl, and diamide (15, 16, 25–28), led to a substantial increase in *Cc*CnoX-GFP expression (Fig. 4). Likewise, *Cc*CnoX was induced when cells were subjected to a 42°C heat shock, a condition that also perturbs protein homeostasis (Fig. 4). Consistently, overexpression of RpoH also increased *Cc*CnoX-GFP production (Fig. 4), indicating that *Cc*CnoX belongs to the RpoH (σ^32^) regulon. Together, these results implicate *Cc*CnoX in the proteostasis network of C. crescentus.

To further explore the role of *Cc*CnoX, we identified proteins that rely on *Cc*CnoX for proper folding. Cultures of a wild-type strain and of a *Cc*CnoX deletion (Δ*CccnoX*) mutant were grown and subjected to thermal stress (42°C for 20 min) when they reached the exponential phase (optical density at 660 nm [OD_660_] of 0.4 [see Materials and Methods]). Cells were collected and the aggregation fraction isolated in order to identify proteins that only aggregated in the absence of *Cc*CnoX. Fifty aggregated proteins were identified via liquid chromatography-tandem mass spectrometry (LC-MS/MS [see Table S1 in the supplemental material]), indicating that *Cc*CnoX provides protection against aggregation to a wide range of cytoplasmic proteins. Interestingly, of the 50 proteins identified, 27 are homologs of known substrates of DnaK or GroEL in E. coli, including GroEL obligate substrates like the fructose-bisphosphate aldolase (Table S1).

10.1128/mBio.01541-18.9 TABLE S1 Substrates of *Cc*CnoX. Download Table S1, PDF file, 0.2 MB. Copyright © 2018 Goemans et al. 2018 Goemans et al. This content is distributed under the terms of the Creative Commons Attribution 4.0 International license.

It is noteworthy that a *CccnoX* deletion mutant did not exhibit any growth phenotype under heat shock (42°C) or oxidative stress (HOCl, H_2_O_2_, or diamide stresses) compared to a wild-type strain. This lack of phenotype can probably be explained by the redundancy of the bacterial proteostatic network.

### *Cc*CnoX functions as a reductase.

Proteins from the CnoX family display an N-terminal Trx fold fused to a C-terminal TPR domain (17). Proteins with a Trx fold usually function as oxidoreductases and rely on a conserved CXXC motif for catalytic activity (13). In *Ec*CnoX, however, this motif is replaced by SXXC. Because the first cysteine of the CXXC motif is absolutely required for activity (13), *Ec*CnoX is unable to function as an oxidoreductase (14). Interestingly, *Ec*CnoX possesses a second cysteine, away from the catalytic site, that enables *Ec*CnoX to form mixed-disulfide complexes with its substrates, thereby protecting their sensitive cysteine residues from overoxidation (14).

**FIG 5:**
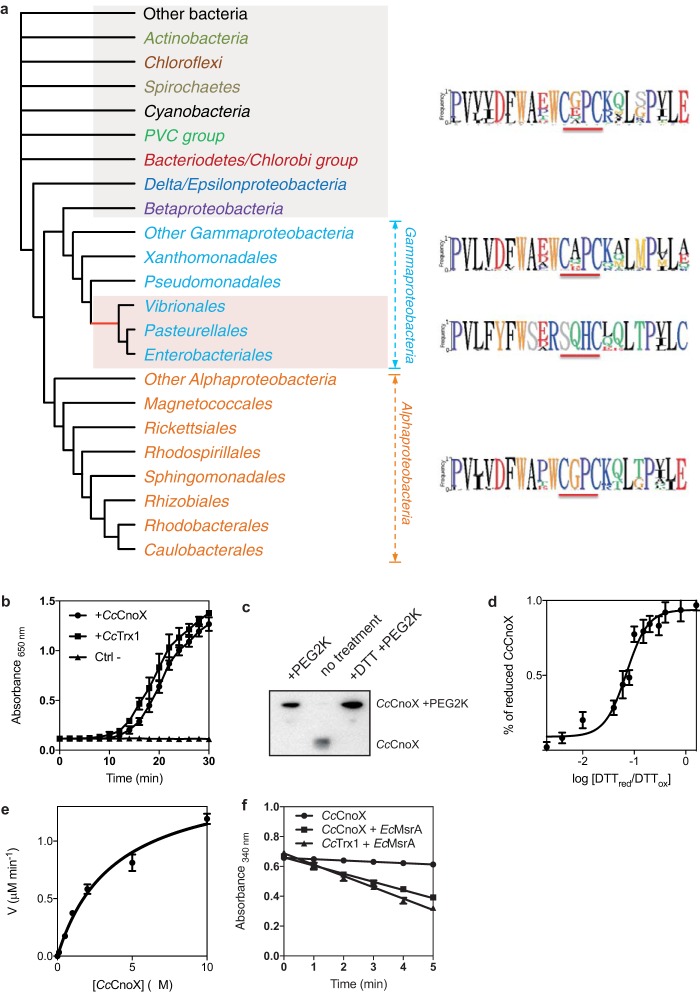
*Cc*CnoX displays reductase activity. (a) CnoX is well conserved among Gram-negative bacteria. This schematic is an unrooted Bayesian phylogenetic tree for CnoX (Materials and Methods). The logo for the Trx catalytic site for each group appears at right. The complete tree is available in the supplemental material (Fig. S1). Red shading indicates bacteria with a CnoX version that lacks the first cysteine of the catalytic site. (b) *Cc*CnoX catalyzes the reduction of insulin by dithiothreitol (DTT). *Cc*Trx1 was used as a positive control, and a sample without Trx (Ctrl−) was used as a negative control. Data are the mean of three independent experiments, and error bars represent the standard deviation. (c) *Cc*CnoX is reduced *in vivo.* Wild-type cultures were harvested with trichloroacetic acid and resuspended in SDS-sample buffer containing the alkylating agent Mal-PEG2k (Materials and Methods). This immunoblot was probed with anti-*Cc*CnoX antibody (Materials and Methods); the molecular weight of *Cc*CnoX increased in the presence of Mal-PEG2k. (d) To calculate the redox potential of *Cc*CnoX, the protein was equilibrated in redox buffers with the indicated ratios of reduced to oxidized DTT. Redox potential was calculated from the ratio of the amounts of oxidized and reduced *Cc*CnoX at equilibrium, determined through AMS trapping experiments (Materials and Methods). The redox potential of *Cc*CnoX is −220 mV. Data are the mean from three independent experiments; error bars denote standard deviation. (e) Reduction of *Cc*CnoX by *Cc*TrxR was monitored by measuring the decrease in absorbance at 340 nm, corresponding to the decrease in reduced NADPH. We measured the initial velocities (*v*) of *Cc*CnoX reduction by *Cc*TrxR to determine the kinetic parameters of the reaction. The *K_m_* of *Cc*TrxR for *Cc*CnoX is 3 μM. Data are the mean from three independent experiments; error bars denote standard deviation. (f) *Cc*CnoX reduces MsrA and DsbDα, known substrates of Trx, as measured by the decrease in absorbance at 340 nm corresponding to the oxidation of NADPH. A sample without substrate was used as a negative control. This experiment was performed in triplicate, and error bars denote standard deviation.

Examination of the *Cc*CnoX sequence revealed that this protein only harbors two cysteine residues, which are present in a WCGPC motif (Fig. 5a). This sequence is typical of Trx proteins that function in a reducing pathway and catalyze disulfide reduction (13). Several results confirmed the reductive function of *Cc*CnoX. First, *Cc*CnoX catalyzed the reduction of insulin by dithiothreitol (DTT) *in vitro* (Fig. 5b), reflecting its ability to function as an oxidoreductase. Second, we assessed the redox status of *Cc*CnoX catalytic cysteines *in vivo* with Mal-PEG2k, a 2-kDa alkylating agent that covalently binds free thiol groups, leading to a band shift on SDS-PAGE. The cysteine residues of *Cc*CnoX were maintained fully reduced *in vivo* (Fig. 5c), implying that *Cc*CnoX functions in a reducing pathway. Third, we measured the redox potential of *Cc*CnoX; redox potential determines the ability of a protein to reduce or to oxidize its substrates. *Cc*CnoX has a redox potential of approximately −220 mV (Fig. 5d), less reducing than that of most Trxs (usually approximately −270 mV [13]). In addition, *Cc*CnoX was efficiently reduced by TrxR (*Cc*TrxR-CCNA_02964) (Fig. 5e), a flavoenzyme that recycles Trx proteins at the expense of NADPH (13). We measured the *K_m_* of *Cc*TrxR for *Cc*CnoX using oxidized *Cc*CnoX as a substrate (*Cc*CnoX was oxidized with diamide) and obtained a value of 3 μM (Fig. 5e), which is in line with previously reported values for the reduction of Trxs by TrxR (29, 30). When recycled by TrxR, *Cc*CnoX also catalyzes the reduction of MsrA and DsbDα, two classical Trx substrates (Fig. 5f). Finally, overexpression of *Cc*CnoX suppressed the essentiality of *Cc*Trx1 (Table 1), a C. crescentus Trx protein with a WCGPC motif that we recently characterized as essential (31) (see Discussion). In combination, these results reveal that *Cc*CnoX is a real Trx oxidoreductase that functions in a classical reducing pathway.

**TABLE 1:** Overexpression of *Cc*CnoX complements a *Cctrx1* deletion

Strain	% of deletion mutants obtained with^*a*^:				
	WT	Low-copy plasmid		High-copy plasmid	
		p-*Cctrx1*	p-*CccnoX*	p-*Cctrx1*	p-*CccnoX*
Δ*Cctrx1*::*tet* mutant	0	48	4	54	42

a This table shows that *Cctrx1* can only be deleted in the presence of an exogenous copy of *Cctrx1* or when *Cc*CnoX is overexpressed. Low expression of *Cc*CnoX is not sufficient to complement a *Cctrx1* knockout. The two-step recombination method used to produce knockouts in C. crescentus leads to the production of 50% deletion mutant and 50% wild-type clones when the gene deleted is not essential. The numbers in the table indicate the percentage of deletion mutants obtained under each tested condition.

**FIG 6:**
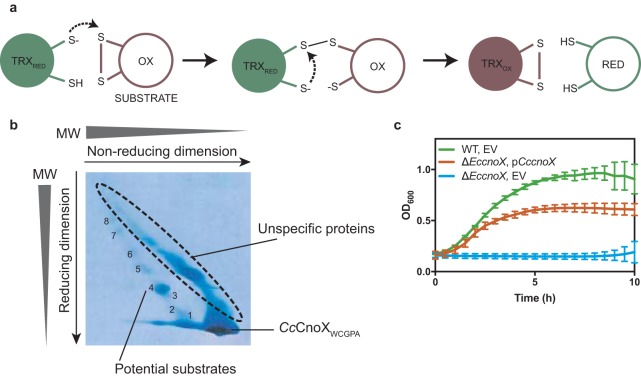
Ninety proteins depend on the reductase activity of *Cc*CnoX *in vivo.* (a) Catalytic cycle of Trxs. The first cysteine of the catalytic motif of Trx performs a nucleophilic attack on an oxidized cysteine in its substrate. This leads to the formation of a mixed-disulfide complex between the Trx and its substrate. Then, the second cysteine of the catalytic site of Trx resolves the disulfide, thereby releasing a reduced substrate. (b) Diagonal gel obtained by thiol trapping experiments. The spots analyzed by mass spectrometry are numbered, and potential candidates are identified in Table S1. Here, we show one representative example of this experiment, which has been conducted in triplicate. (c) Growth curves of wild-type cells harboring an empty vector (EV [green]), Δ*EccnoX* cells plus EV [blue]), and Δ*EccnoX* cells expressing *Cc*CnoX in *trans* (orange) show that *Cc*CnoX partially complements an *EccnoX* deletion under HOCl stress. Cells were grown in M9 + glucose medium after addition of 2 mM HOCl. The OD_600_ was measured for 10 h. This graph shows the mean from three independent experiments; error bars are standard errors of the mean (SEM).

To further probe the importance of the redox activity of *Cc*CnoX in the proteostasis network of C. crescentus, we identified proteins that depend on its Trx activity for redox control. The catalytic cycle of Trxs, which is well understood (13), starts with the first cysteine residue of the WCGPC motif performing a nucleophilic attack on an oxidized cysteine in a substrate protein. This attack leads to the formation of a mixed-disulfide intermediate between the Trx and its substrate, which is resolved via nucleophilic attack by the second cysteine of the catalytic motif, leading to the release of a reduced substrate and an oxidized Trx (Fig. 6a) (13). A powerful approach to identify Trx substrates consists in trapping mixed-disulfide intermediates by mutating the second cysteine of the WCGPC motif to an alanine. Dissociation of the intermediate is prevented, which stabilizes complexes between Trx and its substrates. Here, a catalytic mutant of *Cc*CnoX with a WCGPA motif (*Cc*CnoX_WCGPA_) and carrying a C-terminal His tag was expressed in a C. crescentus wild-type strain (Materials and Methods). The mixed-disulfide complexes formed between *Cc*CnoX_CXXA_ and its substrates were purified via affinity chromatography and analyzed by two-dimensional gel electrophoresis, in which the first dimension was nonreducing and the second reducing (Materials and Methods). Thus, proteins that were not linked to *Cc*CnoX_WCGPA_ by a disulfide bond were separated according to molecular weight in both dimensions, migrating on a diagonal in the second dimension. In contrast, proteins that were in a mixed-disulfide complex with *Cc*CnoX_WCGPA_ were separated from it in the second dimension and appeared off the diagonal (Fig. 6b). The latter set of proteins was identified via tandem mass spectrometry (MS/MS). Ninety cysteine-containing proteins for which peptides were found off the diagonal in at least two of three independent experiments were considered to be putative *Cc*CnoX substrates (Table S1). These proteins included 14 proteins known to be Trx1 substrates in E. coli (32) (Table S1). Interestingly, two proteins (cysteine desulfhydrase/selenocysteine lyase and fructose-bisphosphate aldolase) that are obligate substrates of GroEL (9) and Trx1 substrates in E. coli (32) were identified as *Cc*CnoX substrates (Table S1), suggesting that in C. crescentus, *Cc*CnoX and GroEL cooperate to keep these proteins folded. Moreover, 10 of the identified proteins were also identified among the proteins that aggregated in cells lacking *Cc*CnoX (Table S1), which supports the idea that *Cc*CnoX protects these proteins from both oxidation and aggregation.

Finally, as *Cc*CnoX and *Ec*CnoX exhibit quite different activities, we probed whether *Cc*CnoX was able to complement the strong growth defect observed in an E. coli *cnoX* deletion (Δ*EccnoX*) mutant upon HOCl treatment. Interestingly, the expression of *Cc*CnoX allowed the strain to grow and survive upon stress, although it never reached the growth levels of a wild-type E. coli strain (Fig. 6c). Because both proteins exhibit similar holdase activities upon HOCl activation, this difference is likely due to the differences in the redox properties of both proteins.

## DISCUSSION

HOCl is an extremely toxic compound produced by the innate immune system to kill bacteria mostly by oxidizing and unfolding their proteins (33, 34). In recent years, several bacterial defense mechanisms against HOCl have been identified, mostly in E. coli (reviewed in reference 26); the extent of this network underscores the importance of protecting bacterial cells from this compound. These protection mechanisms include HOCl-sensitive transcription factors that control cellular responses to HOCl insults (like HypT or NemR), chaperone holdases that help HOCl-exposed cells to cope with protein aggregation (Hsp33 and polyphosphate), and rescue enzymes that catalyze the reduction of HOCl-oxidized amino acids in proteins (thioredoxins, glutaredoxins, and methionine sulfoxide reductases) (26). Interestingly, homologs of these proteins are present in many bacteria, including some that are unlikely to encounter HOCl in their environment (Fig. 5a). Although we cannot exclude the possibility that additional sources of HOCl remain to be discovered, proteins that play a role in HOCl protection in certain bacteria may play different roles in other species. Here, we interrogated this hypothesis.

In this study, we investigated the homolog of CnoX expressed by C. crescentus, a non-infectious bacterium commonly found in ponds and streams, aquatic environments lacking obvious sources of HOCl. In contrast to its E. coli counterpart, which needs to be activated by HOCl to function as a chaperone (14), we established that *Cc*CnoX exhibits constitutive holdase activity (Fig. 1), most likely because it displays a more hydrophobic surface than *Ec*CnoX (Fig. 2). As a result, whereas *Ec*CnoX only protects cells against HOCl-induced aggregation (14), *Cc*CnoX is less restrictive and permanently contributes to proteostasis in C. crescentus. The aggregation of proteins under heat stress in cells lacking *Cc*CnoX (Table S1), a condition under which *Cc*CnoX levels normally increase (Fig. 4), demonstrates the physiological relevance of its chaperone activity. Thus, within the family of CnoX proteins, only certain proteins (such as *Ec*CnoX) have evolved to provide protection against HOCl stress. Future work should determine whether this specificity applies to other HOCl-activated proteins, such as the E. coli chaperone Hsp33, which is turned into a holdase by HOCl via the oxidation of a cysteine based-redox switch (35).

We previously established *Ec*CnoX as the first holdase to cooperate with GroEL/ES (14). Here we established that this feature is conserved in *Cc*CnoX (Fig. 3), which suggests that *Ec*CnoX and *Cc*CnoX possess structural properties, possibly shared by all CnoX homologs, that enable them to cooperate with GroEL/ES. Interestingly, it has previously been reported that *Ec*CnoX directly interacts with GroEL (17), perhaps driving the functional cooperation between these two proteins. However, in those *in vitro* experiments, GroEL was pulled down with *Ec*CnoX when the holdase was immobilized on cyanogen bromide-derivatized resin (17); since *Ec*CnoX was perhaps partially unfolded, the physiological relevance of that finding is questionable. Further work should be carried out to confirm that GroEL and *Ec*CnoX interact. If they do, this interaction should be characterized in order to determine its molecular importance for the GroEL-*Ec*CnoX cooperation and to reveal the conservation of this interaction among CnoX homologs.

Another remarkable feature of *Cc*CnoX is its oxidoreductase activity. *Cc*CnoX should be considered a member of the Trx family for at least three reasons. First, it harbors a WCGPC catalytic motif (Fig. 5), the hallmark of Trx proteins in all living organisms (13). Second, *Cc*CnoX is a substrate for TrxR (Fig. 5e), the flavoenzyme that recycles Trx at the expense of NADPH. Third, *Cc*CnoX reduces common Trx substrates (Fig. 5f). Thus, *Cc*CnoX possesses all the attributes necessary to function as a general oxidoreductase in C. crescentus. The identification of 90 redox substrates of *Cc*CnoX (Table S1) agrees with this idea. We previously reported that *Cc*Trx1, the only single-domain Trx expressed by C. crescentus, is essential—probably because it is responsible for the recycling of ribonucleotide reductase (31), the essential enzyme that catalyzes the reduction of ribonucleotides to their corresponding deoxyribonucleotides (36). Since *Cc*CnoX does not rescue the growth of the nonviable Δ*Cctrx1* mutant unless overexpressed (Table 1), the two proteins likely have different substrate specificities.

C. crescentus appears to have a rather limited set of antioxidant enzymes, encoding, in addition to *Cc*CnoX and *Cc*Trx1, only one dithiol-glutaredoxin (*Cc*Grx1 [CCNA_00872]). Dithiol-glutaredoxins are reducing enzymes that cooperate with Trxs in keeping cytoplasmic proteins reduced (37). In comparison, E. coli encodes two single-domain Trxs and three dithiol-glutaredoxins (38). In addition, in C. crescentus, *Cc*Trx1 is thought to play a rather specific role, with expression levels that vary throughout the cell cycle: while the expression of *Cc*Trx1 is induced before DNA replication initiation, the protein is actively degraded by the ClpXP protease in predivisional cells (31). In contrast, the expression of *Cc*CnoX is not cell cycle regulated (data not shown). Thus, both the limited number of antioxidant enzymes expressed by C. crescentus and the fact that *Cc*Trx1 is only expressed during a specific time window of the cell cycle suggest that the oxidoreductase activity of *Cc*CnoX is important for intracellular redox homeostasis in this bacterium.

Important information can be obtained from the phylogenetic analysis of CnoX proteins (Fig. 5a). First, the presence of a strictly conserved WCGPC motif in all CnoX homologs expressed by alphaproteobacteria is a strong indication that these homologs are also members of the Trx family and suggests that these proteins play a role similar to that of *Cc*CnoX in redox homeostasis. It is interesting to note, however, that in a large number of bacterial species, including some gamma-proteobacteria, cyanobacteria, and spirochetes, CnoX displays a WCXPC motif instead of a WCGPC motif (Fig. 5a). Because the identity of the residues that are flanked by the two catalytic cysteine residues has been shown to modulate the redox properties of oxidoreductases with a Trx fold (13), it thus remains to be determined whether these proteins function in a reducing pathway like *Cc*CnoX. Future research will also reveal whether *Cc*CnoX serves as the prototype for the large group of CnoX homologs with a WCG/XPC catalytic motif. Finally, it is striking that only a small subgroup of gamma-proteobacteria, which includes E. coli, possesses a version of CnoX with an SXXC motif. Remarkably, all CnoX homologs with an SXXC motif contain a second, conserved cysteine (not shown) that has been shown in *Ec*CnoX to be required for the formation of mixed-disulfide complexes between *Ec*CnoX and its substrates. This suggests that CnoX has lost the ability to function as a reductase in these organisms, while gaining the capacity to protect its substrates from overoxidation.

An interesting question is why a protein with a domain exhibiting oxidoreductase activity juxtaposed to a domain with holdase function has evolved. We think that combining the ability to reduce a disulfide bond with the ability to prevent protein aggregation offers a great advantage to bacteria. Under oxidative stress, nonnative disulfides form, which can lead to protein misfolding and aggregation. However, the major cytoplasmic foldases GroEL and DnaK are unable to efficiently refold oxidized proteins and lack the ability to reduce oxidized substrates. Therefore, prior to refolding, oxidized substrates need to be reduced. Although this reduction could be carried out by an independent Trx, having a Trx protein fused to a domain with holdase function appears to be an elegant solution to the problem: *Cc*CnoX is a Swiss Army knife that prevents protein aggregation, reduces disulfides when they form, and transfers substrates to both GroEL/ES and DnaK/J/GrpE.

In our previous study, we established *Ec*CnoX as a protein folding factor that is key for cell survival during HOCl stress in E. coli. Our experiments demonstrated that *Ec*CnoX provides an original solution to two threats that proteins face under HOCl stress: aggregation and overoxidation (14). Indeed, *Ec*CnoX combines holdase activity, by which it prevents protein aggregation, with the ability to form mixed-disulfide complexes with client proteins. Using this second function, *Ec*CnoX protects sensitive cysteine residues in its substrates from irreversible oxidation, which could otherwise interfere with refolding and/or block reactivation (14). We proposed that *Ec*CnoX was the first identified member of a novel protein family, the chaperedoxins, whose members combine a chaperone holdase function with a redox protective function (14). In this study, we investigated the CnoX homolog expressed by C. crescentus, a non-pathogenic bacterium that is unlikely to be exposed to HOCl in its environment. We found that *Cc*CnoX has two major characteristics that differentiate it from *Ec*CnoX. First, whereas *Ec*CnoX needs to be activated as a holdase by HOCl, *Cc*CnoX is a constitutive chaperone. *Cc*CnoX holdase activity is stimulated by HOCl, at least *in vitro*, but the physiological relevance of this observation is currently missing. Second, *Cc*CnoX does not protect its substrates from overoxidation by forming stable mixed-disulfide complexes with them, but rather functions as an oxidoreductase that contributes to redox homeostasis. However, despite these differences, *Cc*CnoX can also be considered a chaperedoxin: a protein with chaperone and redox functions. Thus, our work reveals how the structural and redox properties of chaperedoxins have been tailored during evolution to meet the needs of their host species. More and more HOCl-activated proteins have been identified, mostly in E. coli. Our work reveals the importance of investigating the activity and function of their homologs in organisms living in bleach-free environments.

## MATERIALS AND METHODS

### Bacterial strains, plasmids, and growth conditions.

The growth conditions used in this study and the protein expression and purification methods, as well as the antibody production methods, are described in Text S1 in the supplemental material. All strains and plasmids are listed in Table S2 in the supplemental material.

10.1128/mBio.01541-18.1 TEXT S1 Growth conditions and protein purifications. Download Text S1, PDF file, 0.1 MB. Copyright © 2018 Goemans et al. 2018 Goemans et al. This content is distributed under the terms of the Creative Commons Attribution 4.0 International license.

10.1128/mBio.01541-18.10 TABLE S2 Strains and plasmids. Download Table S2, PDF file, 0.04 MB. Copyright © 2018 Goemans et al. 2018 Goemans et al. This content is distributed under the terms of the Creative Commons Attribution 4.0 International license.

### Thermal aggregation of citrate synthase.

The following experiments were performed according to protocols published previously (20). Briefly, 2 μM CS (Sigma-Aldrich) was incubated in 40 mM HEPES-KOH (pH 7.5) at 43°C in the presence or absence of various concentrations of *Cc*CnoX or *Cc*CnoX_HOCl_. Aggregation was monitored by measuring light scattering at 360 nm (Varian Cary 50 UV-Vis spectrophotometer).

### Nile red-based hydrophobicity assay.

*Cc*CnoX and *Ec*CnoX were incubated in the presence of 2 mM HOCl at room temperature for 10 min. HOCl was subsequently removed using a Nap-5 gel filtration column (Sigma-Aldrich). *Cc*CnoX, *Ec*CnoX, *Cc*CnoX_HOCl_, and *Ec*CnoX_HOCl_ (20 μM) were mixed in 40 mM HEPES-KOH (pH 7.5) containing 0.2 μM Nile red (Sigma-Aldrich) (dissolved in dimethyl sulfoxide). Fluorescence was monitored (Biotek Synergy H1 hybrid microplate reader) with 550 nm and 630 nm as the excitation and emission wavelengths, respectively.

### *Cc*CnoX structure modeling.

The structural model of *Cc*CnoX was built using SWISS-MODEL (39–42) with *Ec*CnoX (PDB no. 3QOU) as a template (30% identity). Hydrophobic patches were detected and measured using the “hydrophobic patches” tool from the Swiss-PdbViewer (43).

### Refolding of CS.

Our protocol was adapted from reference 20. Briefly, 15 μM CS was unfolded via dilution in 4 M guanidine hydrochloride in TE buffer (50 mM Tris-HCl [pH 8.0], 2 mM EDTA) and incubated at 16°C for 2 h. Unfolded CS was diluted 1:160 (final concentration, 75 nM) in 40 mM HEPES-HOH (pH 8.0), 10 mM KCl, and 2 mM Mg-ATP in the absence or presence of 0.45 μM *Cc*CnoX. After 20 min of incubation at 25°C, the DnaK/J/GrpE refolding system from C. crescentus (respectively, 0.4, 0.16, and 0.4 μM) or the GroEL_14_ES_7_ refolding system from C. crescentus (respectively, 0.15 and 0.5 μM) was added to the refolding solution and incubated for 2 h at 25°C. Four microliters of this mixture was added to 200 μl of a mixture of 100 μM oxaloacetic acid, 100 μM 5,5′-dithiobis-(2-nitrobenzoic acid), and 160 μM acetyl coenzyme A (acetyl-CoA) in TE buffer in a 96-well plate. Absorbance at 412 nm was monitored for 1 min (Biotek Synergy H1 hybrid microplate reader). The initial slope was used as a proxy to measure the activity of CS.

### Induction assay.

Strain CG129 (Table S2) was grown in M2G medium until it reached an OD_660_ of 0.4. Cells were treated with 20 μM CdCl_2_, 500 μM HOCl, 2 mM H_2_O_2_, and 2 mM diamide or heat shocked at 42°C. After 30 min, the fluorescence (580/620 nm) was measured and normalized by the OD measurement.

### Isolation of aggregated proteins.

Our protocol was adapted from reference 44 and is described in Text S2.

10.1128/mBio.01541-18.2 TEXT S2 Isolation of aggregated proteins. Download Text S2, PDF file, 0.1 MB. Copyright © 2018 Goemans et al. 2018 Goemans et al. This content is distributed under the terms of the Creative Commons Attribution 4.0 International license.

### Data set construction and phylogenetic analyses.

The phylogenetic analyses that were carried out to build the tree presented in Fig. 5 and Fig. S1 are described in Text S3 in the supplemental material.

10.1128/mBio.01541-18.3 TEXT S3 Phylogenetic analyses. Download Text S3, PDF file, 0.1 MB. Copyright © 2018 Goemans et al. 2018 Goemans et al. This content is distributed under the terms of the Creative Commons Attribution 4.0 International license.

### Insulin reduction assay.

Insulin reduction was assayed as described previously (45). Briefly, 150 μM insulin and 10 μM Trx (purified *Cc*Trx1 or *Cc*CnoX) were mixed in 100 mM KPi (pH 7.0) and 1 mM EDTA. The reaction was initiated by adding a final concentration of 0.8 mM dithiothreitol. Insulin reduction was monitored with a spectrophotometer (Varian Cary 50 UV-Vis) at 650 nm.

### *In vivo* redox state determination.

The *in vivo* redox state of *Cc*CnoX was assessed using Mal-PEG2k trapping experiments. Briefly, bacteria were cultured at 30°C in M2G medium until they reached an OD_660_ of 0.4. Proteins were then trichloroacetic acid (TCA) precipitated with 10% cold TCA and resuspended in 30 µl of a mixture of 20 mM Mal-PEG2k (Sigma-Aldrich), 0.1% SDS, 10 mM EDTA, and 50 mM Tris-HCl (pH 7.5). Samples were protected from light and incubated for 45 min at 37°C. Positive controls consisted of protein pellets treated with 50 mM dithiothreitol in the presence of 200 mM Tris (pH 8) and 1% SDS. Protein was detected via immunoblotting with an anti-*Cc*CnoX primary antibody (1:5,000 dilution, rabbit serum, CER group). Chemiluminescence was quantified with a GE ImageQuant LAS4000 camera (GE Healthcare Life Sciences).

### Measurement of redox potential.

*Cc*CnoX (1 μM) was incubated overnight at room temperature in a mixture of 50 mM KPi (pH 7.0) and 0.1 mM EDTA, with various ratios of oxidized and reduced dithiothreitol. The protein was precipitated with trichloroacetic acid (TCA) at a final concentration of 10%. After 20 min of incubation on ice, samples were centrifuged at 16,100 × *g* for 5 min at 4°C, and pellets were washed with cold acetone. After a second centrifugation, pellets were dried and resuspended in nonreducing SDS sample buffer (2% SDS, 10% glycerol, 0.002% bromophenol blue, 0.062 M Tris-HCl [pH 6.8]) containing 20 mM 4-acetamido-4′-maleimidylstilbene-2,2′-disulfonic acid (AMS). After 45 min at 37°C, samples were loaded for SDS-PAGE (NuPAGE Bis-Tris, 12% [Thermo Fisher Scientific]) and colored with PageBlue protein staining solution (Thermo Fisher Scientific). Fractions of oxidized and reduced proteins were determined with ImageJ, and the redox potential was calculated as previously described (46).

### Measurement of *K_m_*.

*Cc*CnoX was oxidized with 40 mM diamide for 30 min at 37°C. Diamide was removed with a desalting column (Nap-5; GE Healthcare). NADPH (200 μM), 1.25 μM *Cc*TrxR, and various concentrations of oxidized *Cc*CnoX (0, 0.1, 0.5, 1, 2, and 5 μM) were mixed, and the absorbance was monitored at 340 nm for 3 min (Varian Cary 50 UV-Vis spectrophotometer). The rate of substrate reduction was calculated as μmol/(min·μg of protein) with the extinction coefficient of NADPH (ε = 6,220 M^−1^cm^−1^) and knowing that for 1 equivalent of NADPH oxidized, 1 equivalent of substrate is reduced.

### NADPH consumption assay.

MsrA and DsbDα (100 μM) were first oxidized with 40 mM diamide for 30 min at 37°C. The diamide was removed with a desalting column (Nap-5; GE Healthcare). NADPH (100 μM), 1.25 μM *Cc*TrxR, and 5 μM *Cc*CnoX were mixed until the slope at 340 nm was stable. Then, 50 μM MsrA or DsbDα was added, and the absorbance was monitored at 340 nm for 3 min (Varian Cary 50 UV-Vis spectrophotometer).

### Complementation assays.

The *Cctrx1* knockout strain was constructed using a classical two-step recombination method described in Text S4 in the supplemental material.

10.1128/mBio.01541-18.4 TEXT S4 Complementation assays. Download Text S4, PDF file, 0.1 MB. Copyright © 2018 Goemans et al. 2018 Goemans et al. This content is distributed under the terms of the Creative Commons Attribution 4.0 International license.

### Thiol trapping and diagonal gel.

The protocols used were adapted from reference 32 and are described in Text S5 in the supplemental material.

10.1128/mBio.01541-18.5 TEXT S5 Thiol trapping and diagonal gel. Download Text S5, PDF file, 0.1 MB. Copyright © 2018 Goemans et al. 2018 Goemans et al. This content is distributed under the terms of the Creative Commons Attribution 4.0 International license.

### Mass spectrometry.

Tryptic peptides were analyzed by liquid chromatography-tandem mass spectrometry (LC-MS/MS) as described in Text S6 in the supplemental material.

10.1128/mBio.01541-18.6 TEXT S6 Mass spectrometry. Download Text S6, PDF file, 0.1 MB. Copyright © 2018 Goemans et al. 2018 Goemans et al. This content is distributed under the terms of the Creative Commons Attribution 4.0 International license.

### Cross-complementation.

Wild-type E. coli or the *EccnoX* mutant either harboring an empty pET22b vector or a plasmid expressing CcCnoX (pET22b::*CccnoX*) were grown in 5 ml M9 plus glucose at 37°C until reaching an OD_600_ of 0.3. Cells were then treated with 2 mM HOCl and transferred to a 96-well plate (Greiner Bio-One). Growth was monitored for 8 h (OD_600_) in a Biotek Synergy H1 hybrid microplate reader.
