# Supplementary material for: The Chaperone and Redox Properties of CnoX Chaperedoxins Are Tailored to the Proteostatic Needs of Bacterial Species
Source: mBio. 2018 Nov 27;9(6):e01541-18. doi: 10.1128/mBio.01541-18 (PMC6282202; doi:10.1128/mBio.01541-18)
Supplement: FIG S1 [file mbo006184194sf1.pdf]

Figure S1- Phylogenetic analysis of *Cc*CnoX

CnoX is well conserved among Gram-negative bacteria. This figure shows a schematic of the unrooted Bayesian phylogenetic tree for *Cc*CnoX.

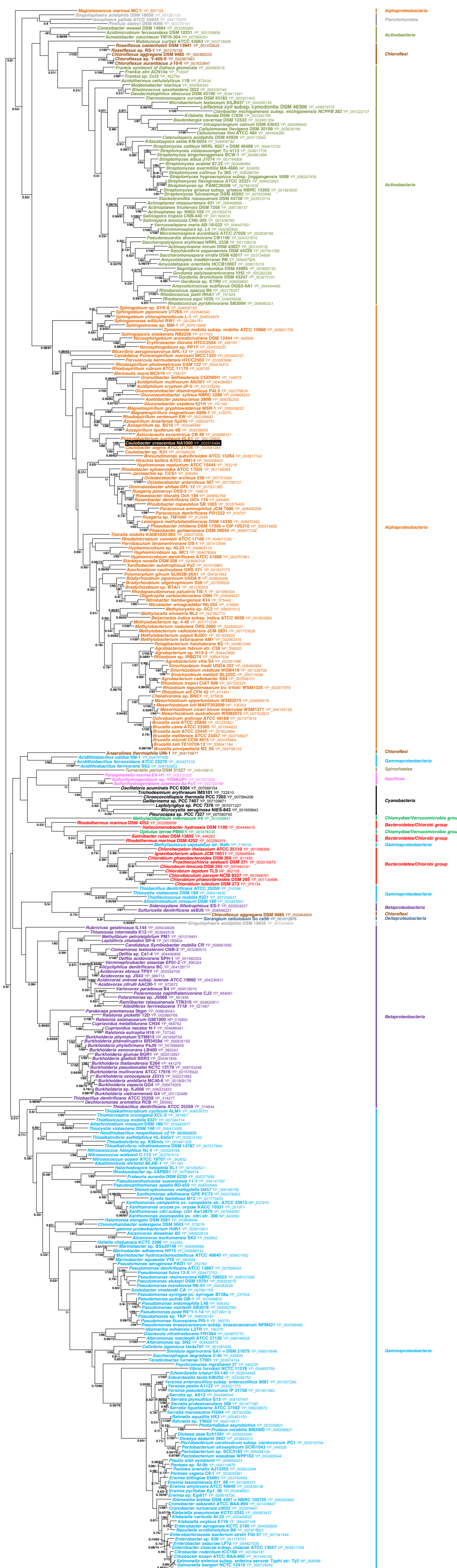

Alphaproteobacteria
Planctomycetes
Actinobacteria
Chloroflexi
Actinobacteria
Alphaproteobacteria
Chloroflexi
Gammaproteobacteria
Spirochaetes
Aquificae
Cyanobacteria
Chlamydiae/Verrucomicrobia group
Bacteroidetes/Chlorobi group
Chlamydiae/Verrucomicrobia group
Bacteroidetes/Chlorobi group
Gammaproteobacteria
Bacteroidetes/Chlorobi group
Gammaproteobacteria
Betaproteobacteria
Chloroflexi
Deltaproteobacteria
Betaproteobacteria
Gammaproteobacteria
0.3
