# Supplementary material for: The Chaperone and Redox Properties of CnoX Chaperedoxins Are Tailored to the Proteostatic Needs of Bacterial Species
Source: mBio. 2018 Nov 27;9(6):e01541-18. doi: 10.1128/mBio.01541-18 (PMC6282202; doi:10.1128/mBio.01541-18)

Figure S2- Holdase experiment in triplicate

A *Cc*CnoX inhibits the aggregation of thermally (43°C) denatured CS (blue, 16:1; orange, 8:1), as measured by light scattering at 360 nm. CS alone is shown in black. This figure shows additional experiments to Figure 4. **B** HOCl-treated *Cc*CnoX inhibits the aggregation of thermally (43°C) denatured CS (orange, 0,5:1; blue, 1:1), as measured by light scattering at 360 nm. CS alone is shown in black. This figure shows additional experiments to Figure 4

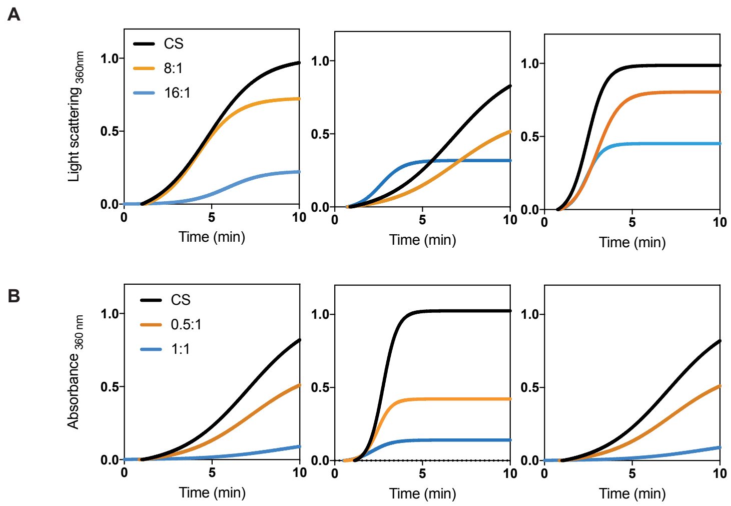

Supplement: FIG S2 [file mbo006184194sf2.pdf]
