# Supplementary material for: The Chaperone and Redox Properties of CnoX Chaperedoxins Are Tailored to the Proteostatic Needs of Bacterial Species
Source: mBio. 2018 Nov 27;9(6):e01541-18. doi: 10.1128/mBio.01541-18 (PMC6282202; doi:10.1128/mBio.01541-18)
Supplement: TABLE S1 [file mbo006184194st1.pdf]

Table S1- Substrates of *Cc*CnoX

This table shows a list of substrates of *Cc*CnoX either identified by aggregation assay or thiol-trapping assay. The "aggregates" column refers to the fold abundance for a given protein in the aggregation fraction of a Δ*cccnoX* compared to WT in the aggregation assay. If the protein was only detected in the absence of *Cc*CnoX, it is marked as Δ*cccnoX*. Proteins identified by thiol-trapping are marked with a X in the "trapping" column and the spot number in which the protein has been identified is indicated for each separate experiment. Proteins that are also known substrates of DnaK in E. coli [1] are marked by a X in the "DnaK" column; Known substrates of *E. coli* GroEL are marked according to Kerner *et al.* classification [2] in the "GroEL" column and known substrates of *E. coli* Trx1 [3] in the "Trx1"column.

CCNA	Accession	Description	Aggregates	Trapping	Exp1 Spot #	Exp2 Spot #	Exp3 Spot #	DnaK	GroEL	Trx 1
01651	B8GVE6	DNA gyrase subunit A	2.8	X	1	/	1		II	
01689	B8GVI4	Inosine-5'-monophosphate dehydrogenase	3					X		
01692	B8GVI7	GMP synthase [glutamine-hydrolyzing]	2.3					X		
03770	B8GVT2	Malate dehydrogenase		X	/	3	2	X		X
03781	B8GVU3	Aconitate hydratase		X	1	7	2			
03833	B8GVZ5	Enoyl-[acyl-carrier-protein] reductase [NADH]		X	2	3	/	X		
03835	B8GVZ7	3-oxoacyl-(Acyl-carrier-protein) synthase		X	1	3	/		I	X
03842	B8GW04	Molybdopterin biosynthesis protein MoeB		X	3	/	7	X		
03871	B8GW33	tRNA uridine 5-carboxymethylaminomethyl modification enzyme MnmG		X	7	/	7			
03873	B8GW35	Putative uncharacterized protein		X	1	5	/			
03876	B8GW38	Transcription termination factor Rho	2.5						II	X
01776	B8GW49	Ribonuclease D	4							
01795	B8GW68	Enolase	3.1	X	3	5	/	X	I	
01799	B8GW72	Pyruvate dehydrogenase E1 component alpha subunit		X	2	/	8	X	I	
01803	B8GW76	dihydrolipoamide acetyltransferase component	3	X	3	5	2	X	I	
01805	B8GW78	Dihydrolipoamide dehydrogenase	2.3	X	1	3	/		I or II	X
02220	B8GWA5	Beta-glucosidase		X	3	3	/			

01858	B8GWD1	Transcriptional regulator, AraC family		X	3	7	/	X		
01943	B8GWL6	Alpha/beta hydrolase		X	4	7	/			
01954	B8GWM7	Ribonuclease E	$\Delta cccnoX$							
01957	B8GWN0	Thiazole biosynthesis protein		X	3	5	/		I	
01961	B8GWN4	Biotin carboxylase	$\Delta cccnoX$						II	X
01969	B8GWP2	Aspartate--tRNA ligase	4					X		
02000	B8GWS3	30S ribosomal protein S2		X	2	5	8		ns	X
02003	B8GWS6	DNA polymerase III subunit alpha		X	3	/	1	X		
02008	B8GWT1	Proline--tRNA ligase	$\Delta cccnoX$					X		
00010	B8GWW7	Chaperone protein DnaK		X	3	1	5		II	
00041	B8GWZ8	Translation initiation factor IF-2	4.8					X	II	
02042	B8GX17	Trigger factor	2.3					X	I	
02047	B8GX22	Glutamine synthetase	2.5					X		X
02050	B8GX25	Dihydroorotase		X	8	/	4	X	II	
02090	B8GX65	Exodeoxyribonuclease III		X	3	5	1			
02134	B8GXA9	Phosphogluconate dehydratase		X	1	5	/			X
00074	B8GXG5	Multifunctional fatty acid oxidation complex subunit alpha	3							
00086	B8GXH7	NAD-specific glutamate dehydrogenase	7.2	X	1	/	8			
00128	B8GXL9	Argininosuccinate synthase	$\Delta cccnoX$					X		
00159	B8GXQ0	DNA gyrase subunit B	3.75							
02231	B8GXY9	ABC transporter ATP-binding protein	8							
02271	B8GY29	Putative uncharacterized protein		X	2	/	3			
00196	B8GY66	3-isopropylmalate dehydratase large subunit		X	/	5	7			
00215	B8GY85	Putative cytosolic protein		X	3	/	8			
00250	B8GYC0	Poly(3-hydroxyalkanoate) depolymerase	$\Delta cccnoX$							
00280	B8GYF0	tRNA pseudouridine synthase A		X	3	6	/		ns	
02362	B8GYQ4	GAF domain-containing protein		X	7	/	6			
02419	B8GYW1	TonB1 protein	$\Delta cccnoX$							

00323	B8GYX4	Low-affinity zinc transport protein	3	X	1	1	1			
00327	B8GYX8	Nitrobenzoate nitroreductase		X	2	5	/			
00340	B8GYZ1	Succinyl-CoA ligase [ADP-forming] subunit beta	2.8							X
00341	B8GYZ2	Succinyl-CoA ligase [ADP-forming] subunit alpha		X	3	3	8			X
00342	B8GYZ3	2-oxoglutarate dehydrogenase E1 component	5.5						II	
00370	B8GZ21	ATP synthase subunit b	3					X		
00398	B8GZ49	Putative uncharacterized protein		X	2	5	1			
00423	B8GZ74	Phosphohydrolase, MutT-nudix family		X	2	5	/			
02553	B8GZM9	ATP-dependent clp protease ATP-binding subunit ClpA	$\Delta cccnoX$					X		
00465	B8GZP6	UDP-galactopyranose mutase		X	3	5	/	X		
00466	B8GZP7	Glycosyltransferase	2.7							
00496	B8GZS7	Threonine--tRNA ligase	$\Delta cccnoX$					X	II	
00505	B8GZT6	Ubiquinol-cytochrome c reductase iron-sulfur subunit		X	5	/	6			
00506	B8GZT7	Cytochrome b		X	2	3	/			
00515	B8GZU6	5-methyltetrahydropteroyltriglutamate--homocysteine methyltransferase		X	3	5	/			
00518	B8GZU9	50S ribosomal protein L25		X	4	7	/			
00520	B8GZV1	Ribose-phosphate pyrophosphokinase		X	3	3	/		II	X
00536	B8GZW7	DNA-directed RNA polymerase subunit beta	3.5					X	II	
00537	B8GZW8	DNA-directed RNA polymerase beta' subunit	3.6	X	1	1	/		II	
00544	B8GZX5	Acetyl-CoA acetyltransferase		X	4	/	8			
02566	B8H023	Membrane alanine aminopeptidase		X	1	/	5			
02575	B8H032	Putative cytosolic protein	2.6							
02596	B8H053	30S ribosomal protein S4		X	8	8	/			
02607	B8H064	Isocitrate dehydrogenase [NADP]		X	1	1	/	X		X
02612	B8H069	Alanine--tRNA ligase		X	1	/	5	X		

02619	B8H076	Stomatin/prohibitin-related protein	4								
02657	B8H0B4	Putative uncharacterized protein		X	3	5	/				
00618	B8H0I4	N-succinylglutamate 5-semialdehyde dehydrogenase		X	1	1	/				
00656	B8H0M2	Type I restriction-modification system methylation subunit		X	3	/	6				
02759	B8H0Z6	Methionine aminopeptidase		X	3	5	/				
00764	B8H1A6	Electron transfer flavoprotein beta-subunit		X	3	7	/				
00778	B8H1C0	GTP-binding protein TypA/BipA	4.7	X	2	3	/	X	II		
00801	B8H1E3	Cytochrome bd-type quinol oxidase, subunit 2 cydB		X	3	6	/	X			
00846	B8H1X2	Proline dehydrogenase/delta-1-pyrroline-5-carboxylate dehydrogenase		X	1	6	/				
00863	B8H1Y9	Xylonolactonase xylC		X	2	3	/				
00893	B8H219	Putative transcriptional regulatory protein	$\Delta cccnoX$								
03040	B8H2I7	Outer membrane pilus secretion channel cpaC		X	6	/	2				
00938	B8H264	Acetyl-CoA acetyltransferase		X	3	7	/				
02979	B8H2C6	Cysteine desulfhydrase/selenocysteine lyase		X	1	1	1	X	III	X	
02981	B8H2C8	Transcriptional regulator, AsnC family		X	6	8	/				
02994	B8H2E1	Carbamoyl-phosphate synthase large chain	$\Delta cccnoX$								
03059	B8H2K6	Putative membrane spanning protein		X	6	1	/				
03090	B8H2N7	Acetyl-coenzyme A carboxylase carboxyl transferase subunit alpha	$\Delta cccnoX$					X	II		
01010	B8H2S1	Histidine ammonia-lyase	$\Delta cccnoX$								
01029	B8H2U0	Alpha-L-rhamnosidase		X	/	3	8				
01087	B8H2Z8	Putative uncharacterized protein		X	6	/	5				
01095	B8H306	Phenylalanine--tRNA ligase beta subunit	3					X			
03108	B8H336	TonB-dependent outer membrane receptor		X	/	3	1				

03162	B8H390	Arginine biosynthesis bifunctional protein ArgJ		X	3	1	/			
01172	B8H3L8	Putative uncharacterized protein		X	5	1	/			
01298	B8H413	Elongation factor Tu		X	1	7	8		ns	
03358	B8H468	Glyceraldehyde 3-phosphate dehydrogenase		X	3	5	/		I	X
01253	B8H481	Dihydrodipicolinate synthase		X	2	/	8	X	III	
01306	B8H4D4	50S ribosomal protein L3		X	2	5	/	X		
01309	B8H4D7	50S ribosomal protein L2		X	2	3	/		ns	X
01312	B8H4E0	30S ribosomal protein S3		X	3	6	6	X	ns	
01320	B8H4E8	30S ribosomal protein S8		X	7	/	5			
01328	B8H4F6	30S ribosomal protein S13		X	8	/	5			X
01330	B8H4F8	DNA-directed RNA polymerase subunit alpha		X	3	5	/	X	I	
03360	B8H4K0	Fructose-bisphosphate aldolase	3						III	X
03371	B8H4L2	Bacterioferritin	$\Delta cccnoX$							
03422	B8H4R3	Oligoendopeptidase F		X	2	2	/			
03462	B8H4V3	Probable glycine dehydrogenase [decarboxylating] subunit 2		X	1	7	/			
03467	B8H4V8	Putative uncharacterized protein		X	2	/	1			
01381	B8H4Z4	Valine--tRNA ligase		X	3	4	/	X		
01406	B8H519	Putative metal-dependent hydrolase	3.1							
01419	B8H532	Serine hydroxymethyltransferase		X	1	1	/		I	X
01427	B8H540	Lipoprotein, SmpA/OmlA family	3							
01440	B8H553	50S ribosomal protein L13		X	6	/	6	X		
03557	B8H5H7	Putative uncharacterized protein		X	4	/	4			
03558	B8H5H8	ATP synthase epsilon chain	4					X		
03560	B8H5I0	ATP synthase subunit beta	2.5	X	3	3	/	X		X
03562	B8H5I2	ATP synthase subunit alpha	2.1	X	1	3	/	X		X
03563	B8H5I3	ATP synthase subunit delta	2.1							
01552	B8H5U9	Polar localization protein tipN	2.8							
01603	B8H600	Aspartate aminotransferase		X	3	5	/	X		

01606	B8H603	Soj/ParA-related ATPase protein		X	4	/	1			
01612	B8H609	Rod shape-determining protein mreB	2.2					X		X
03643	B8H647	Succinate dehydrogenase membrane anchor subunit		X	3	/	3			
03681	B8H685	ABC transporter ATP-binding protein	Δ*cccnoX*							
03677	B8H681	CoA-transferase family III protein		X	6	/	6			
03683	B8H687	Maleylpyruvate isomerase		X	4	/	3			
03689	B8H693	Alanine dehydrogenase		X	3	6	/			
03695	B8H699	Aldehyde dehydrogenase		X	3	/	3		III	
03711	B8H6B5	Ribosome-associated factor Y		X	3	7	/			

1 Calloni, G. *et al.* DnaK functions as a central hub in the E. coli chaperone network. *Cell Rep* **1**, 251-264, doi:10.1016/j.celrep.2011.12.007 (2012).

2 Kerner, M. J. *et al.* Proteome-wide analysis of chaperonin-dependent protein folding in Escherichia coli. *Cell* **122**, 209-220, doi:10.1016/j.cell.2005.05.028 (2005).

3 Arts, I. S., Vertommen, D., Baldin, F., Laloux, G. & Collet, J. F. Comprehensively Characterizing the Thioredoxin Interactome In Vivo Highlights the Central Role Played by This Ubiquitous Oxidoreductase in Redox Control. *Mol Cell Proteomics* **15**, 2125-2140, doi:10.1074/mcp.M115.056440 (2016).
