# Supplementary material for: The Chaperone and Redox Properties of CnoX Chaperedoxins Are Tailored to the Proteostatic Needs of Bacterial Species
Source: mBio. 2018 Nov 27;9(6):e01541-18. doi: 10.1128/mBio.01541-18 (PMC6282202; doi:10.1128/mBio.01541-18)
Supplement: TEXT S1 [file mbo006184194s1.pdf]

Growth conditions

Caulobacter crescentus cells were grown in M2G medium (6.1 mM Na_2HPO_4, 3.9 mM KH_2PO_4, 9.3 mM NH_4Cl, 0.5 mM $MgSO_4$, 10 µM $FeSO_4$, 0.5 mM $CaCl_2$, and 0.2% glucose) at 30 °C unless indicated otherwise. All experiments were performed at mid-log phase ($OD_{660\ nm}$=0.3-0.4). *Escherichia coli* cells were used for protein expression and purification and were grown in LB at 37 °C. For plasmid maintenance in *E. coli*, ampicillin was used at 100 µg/mL. For plasmid maintenance in *C. crescentus*, kanamycin (5 µg/mL), gentamycin (2 µg/mL), and oxytetracyclin (1 µg/mL) were used. All plasmids were cloned through restriction ligation using the Thanbichler plasmid collection (35).

Protein expression, purification, and antibody production

*Cc*CnoX was purified without a tag from strain CG197 harboring a pET22b(+) plasmid (Novagen). After an overnight preculture, cells were diluted in 3 L of LB containing 100 µg/mL ampicillin and grown at 37 °C in a shaking incubator. At mid-log phase, expression was induced with 1 mM IPTG for 3 h. Cells were harvested, resuspended in Tris 50 mM [pH 8.0], and lysed with a french press. After a 40-min centrifugation at 23,000 x *g* and 4 °C, the supernatant was loaded onto a 5-mL Q-Sepharose HP column (GE Healthcare). Proteins were eluted with a 0-40 % 1M NaCl gradient in Tris 50 mM [pH 8.0]. Proteins were subjected to a second step of purification using a HiLoad S75 16/60 gel filtration column (GE Healthcare) and eluted in 10 mM HEPES-KOH [pH 8.0], and 100 mM NaCl.

*Cc*TrxR, *Cc*DnaK, *Cc*DnaJ, *Cc*GrpE, and *Cc*GroES were respectively purified from strains CG56, CG196, CG190, CG193, and CG177 carrying a pET plasmid (**Table S2**). The proteins harbored a His-Tag at the C-terminus. After overnight preculture, cells were diluted in 3 L of LB containing 100 µg/mL ampicillin and grown at 37 °C in a shaking incubator. At mid-log

phase, protein expression was induced with 1 mM IPTG for 3 h. Cells were harvested, resuspended in 50 mM NaPi [pH 8.0], 300 mM NaCl and lysed with a french press. After a 40-min centrifugation at 23,000 x *g* and 4 °C, the supernatant was loaded onto a 5-mL HisTrap HP column (GE Healthcare). Proteins were eluted with a gradient containing 0-40% buffer B (50 mM NaPi [pH 8.0], 300 mM NaCl, and 300 mM imidazole). Samples were subjected to a second step of purification with a HiLoad S75 16/60 gel filtration column (GE Healthcare) and eluted in 10 mM HEPES-KOH [pH 8.0], 100 mM NaCl.

*Cc*GroEL was purified without tag from CG189 (**Table S2**). After overnight culture, cells were grown in 3 L of LB until mid-log phase and expression was induced with 1 mM IPTG. After 3 h, cells were collected and resuspended in buffer containing 50 mM Tris-HCl [pH 7.4], 1 mM EDTA, and 1 mM dithiothreitol. After lysis with a French press, the sample was ultra-centrifuged (184,246 x *g* for 1 h at 4 °C), loaded onto a 5-mL DEAE-sepharose HP column (GE Healthcare), and eluted with a gradient (0-50%) of 50 mM Tris-HCl [pH 7.4], 1 mM EDTA, 1 mM dithiothreitol, and 1M NaCl. The second purification step consisted of gel filtration (HiLoad S200 16/600 column; GE Healthcare) in buffer containing 10 mM HEPES-KOH [pH 8], 100 mM NaCl, and 1 mM dithiothreitol. Finally, the sample was loaded onto a 5-mL Q-Sepharose HP column (GE Healthcare) using the conditions described for the first step.

CcTrx1 was purified as described previously (27).
