# Supplementary material for: The Chaperone and Redox Properties of CnoX Chaperedoxins Are Tailored to the Proteostatic Needs of Bacterial Species
Source: mBio. 2018 Nov 27;9(6):e01541-18. doi: 10.1128/mBio.01541-18 (PMC6282202; doi:10.1128/mBio.01541-18)
Supplement: TABLE S2 [file mbo006184194st2.pdf]

Table S2- Strains and plasmids

Strains	Features or relevant genotype	Source and notes
MG1655	WT *E. coli*	From Jim Bardwell
JN17	CB15N	From Xavier De Bolle
CG69	CB15N *ybbN::tetRA*	This study
CG126	CG69 pBXMCS2-CcybbNcxxa	This study
CG129	CB15N *ybbN*::*ybbN-mCherry* (pCHYC-2)	This study
FB18	CB15N pBXMCS4-*CcybbN*	This study
FB44	CB15N D*trx1* pBXMCS4-*CcybbN*	This study
CG197	BL21 pET22b-*CcybbN* (no tag)	This study
CG56	BL21 pET23a-*CctrxR* (HisTag)	This study
CG196	BL21 pET22b-*CcdnaK*	This study
CG190	BL21 pET22b-*CcdnaJ*	This study
CG193	BL21 pET22b-*CcgrpE*	This study
CG189	BL21 pET22b-*CcgroEL*	This study
CG177	BL21 pET22b-*CcgroES*	This study
