# Supplementary material for: The Chaperone and Redox Properties of CnoX Chaperedoxins Are Tailored to the Proteostatic Needs of Bacterial Species
Source: mBio. 2018 Nov 27;9(6):e01541-18. doi: 10.1128/mBio.01541-18 (PMC6282202; doi:10.1128/mBio.01541-18)
Supplement: TEXT S2 [file mbo006184194s2.pdf]

Isolation of aggregated proteins

Cells were grown in PYE broth (containing 0.2% Bacto Peptone, 0.1% yeast extract, 1 mM $MgSO_4$, and 0.5 mM $CaCl_2$) until $OD_{660\ nm}$=0.5 and then stressed for 30 min at 42 °C. Cells were cooled on ice for 15 min and centrifuged at 5000 x *g* for 10 min at 4 °C. Pellets were resuspended in 120 μL of fresh fractionation buffer (10 mM KPi [pH 6.5], 1 mM EDTA, 20% sucrose, and 1 mg/mL lysozyme) and kept on ice for 30 min. Buffer B (1080 μL; 10 mM KPi [pH 6.5], 1 mM EDTA) was added to the sample before sonication. Cells were centrifuged at 2000 x *g* for 15 min at 4 °C to remove intact cells. The supernatant was transferred into a clean tube and centrifuged at 14,000 x g for 20 min. The pellet was kept and frozen at -80 °C and stored overnight. The next day, pellets were resuspended in 1 mL of buffer B and briefly sonicated before being centrifuged at 14,000 x g for 20 min at 4 °C. The pellet was washed with 960 μL of buffer B and 240 μL of 10% NP40 detergent and centrifuged at 14,000 x g for 30 min at 4 °C. The washing step was repeated a second time. Finally, the clean pellet was precipitated with TCA and dried for mass spectrometry.
