# Supplementary material for: The Chaperone and Redox Properties of CnoX Chaperedoxins Are Tailored to the Proteostatic Needs of Bacterial Species
Source: mBio. 2018 Nov 27;9(6):e01541-18. doi: 10.1128/mBio.01541-18 (PMC6282202; doi:10.1128/mBio.01541-18)
Supplement: TEXT S3 [file mbo006184194s3.pdf]

Dataset construction and phylogenetic analyses

A non-redundant local protein database containing 1,342 complete prokaryotic proteomes available from NCBI (http://www.ncbi.nlm.nih.gov/) as of July 30, 2014 was built. This database was queried with BLASTP (42) (default parameters, using CCNA_00109 (YP_002515484) of *Caulobacter crescentus* NA1000 as a seed). Homologous and non-homologous sequences were distinguished through visual inspection of each BLASTP output (no arbitrary cut-off E-value or score). In order to ensure that we did not overlook divergent CnoX (YbbN) proteins, iterative BLASTP queries were performed using homologs identified at each step as new seeds. The retrieved sequences were aligned using MAFFT v7.045b (43). Each alignment was visually inspected and manually refined when necessary using the ED program from the MUST package. Regions where the homology between amino-acid positions was doubtful were removed with BMGE (BLOSUM30 similarity matrix) (44). Preliminary phylogenetic analysis was performed using FastTree v.2 (45) using a gamma distribution with four categories. Based on the resulting tree, the subfamily containing the CnoX sequence with Trx and TPR domains was identified and selected for further phylogenetic investigations. Corresponding sequences were realigned using MAFFT version 7 (43). The resulting alignment was trimmed with BMGE as described above. Maximum likelihood trees were computed using PhyML version 3.1 (46) with the Le and Gascuel model (amino-acid frequencies estimated from the dataset) and a gamma distribution (four discrete categories of sites and an estimated alpha parameter) to account for variations in evolutionary rate across sites. Branch robustness was estimated with the non-parametric bootstrap procedure implemented in PhyML (100 replicates of the original dataset with the same parameters). Bayesian inferences were performed using MrBayes 3.2 with a mixed model of amino-acid substitution including a gamma distribution (four discrete categories). MrBayes was run with four chains for 1 million

generations and trees were sampled every 100 generations. To construct the consensus tree, the first 2000 trees were discarded as “burn in”.
