# Supplementary material for: The Chaperone and Redox Properties of CnoX Chaperedoxins Are Tailored to the Proteostatic Needs of Bacterial Species
Source: mBio. 2018 Nov 27;9(6):e01541-18. doi: 10.1128/mBio.01541-18 (PMC6282202; doi:10.1128/mBio.01541-18)
Supplement: TEXT S4 [file mbo006184194s4.pdf]

Complementation assays

The *cctrx1* knock-out strain was constructed using a two-step recombination method. The upstream and downstream regions of *cctrx1* were amplified (upstream: 5'-gaggatatctggccatggccagcgcggcgtttg-3' and 5'-ctcgctagccgcaacggtgctcatggtctgctc-3'; downstream: 5'-gaggctagcggcgtccaggccgcctaagcggc-3' and 5'-ctctccggatccagctggagccggccc-3') from CB15N chromosomal DNA and cloned together into pNPTS138, to create an in-frame deletion. This plasmid was transformed into *C. crescentus* via electroporation, plated on PYE-Kan (final kanamycin concentration, 20 μg/mL) to select for plasmid integration, and incubated at 30 °C for 2 days. Several integrants were inoculated into PYE (0.2% w/v Bacto Peptone- BD Biosciences, 0.1% w/v yeast extract- BD Biosciences, 1 mM $MgSO_4$, and 0.5 mM $CaCl_2$) and grown at 30 °C to allow the second recombination to occur. Cells were plated on PYE containing 3% sucrose to select for cells that lost the plasmid (*sacB* counter-selection). Plates were incubated at 30 °C for 2 days. The resulting clones were patched onto PYE and PYE-Kan plates to select clones that lost the plasmid, not the ones with an inactivated copy of *sacB.* The knock-out construct was verified with PCR using primers (5'-gaggatatctggccatggccagcgcggcgtttg-3' and 5'-ctctccggatccagctggagccggccc -3') upstream and downstream of *cctrx1.*
