# Supplementary material for: The Chaperone and Redox Properties of CnoX Chaperedoxins Are Tailored to the Proteostatic Needs of Bacterial Species
Source: mBio. 2018 Nov 27;9(6):e01541-18. doi: 10.1128/mBio.01541-18 (PMC6282202; doi:10.1128/mBio.01541-18)
Supplement: TEXT S5 [file mbo006184194s5.pdf]

Thiol-trapping and diagonal gel

Strain CG126 (**Table S2**) was grown in 1 L of PYE at 30 °C until $OD_{660\ nm}$=0.3. Expression of *Cc*$CnoX_{WCGPA}$ was induced by adding a final concentration of 0.3% xylose. After 2 h, the culture was precipitated with 10% TCA and incubated at 4 °C overnight. Cells were centrifuged at 11,325 x *g* for 45 min at 4 °C. Pellets were resuspended in 50 mL of 5% cold TCA and centrifuged at 17,696 x *g* for 20 min at 4 °C. Proteins were resuspended in 25 mL of 100 mM NaPi [pH 8.0], 300 mM NaCl, 0.3% SDS, 8 M urea, and 10 mM iodoacetamide to prevent further rearrangements of disulfide bonds. The lysate was homogenized by shaking for 20 min and centrifuged at 23,708 x *g* for 45 min at room temperature. The cleared lysate was diluted three times in buffer A (50 mM NaPi [pH 8.0], 300 mM NaCl, and 0.3% SDS) and loaded onto a 1-mL HisTrap FF column (GE Healthcare), previously equilibrated with buffer A. After washing with buffer A, proteins were eluted with a linear gradient from 0 to 300 mM imidazole in buffer A. Only one peak eluted from the column. This fraction was concentrated to 1.5 mL using a Vivaspin Turbo 15 device (Sartorius) and proteins were resolved on SDS-PAGE under non-reducing conditions (first dimension). The gel lane was cut, incubated in 20 mL of buffer containing 10% SDS, 0.3 M Tris [pH 7.5], 100 mM dithiothreitol, and 50% glycerol for 1 h, before being placed on top of a second SDS-PAGE gel. After electrophoresis, proteins were visualized with PageBlue Protein Staining Solution (Thermo Scientific).
