# Supplementary material for: The Chaperone and Redox Properties of CnoX Chaperedoxins Are Tailored to the Proteostatic Needs of Bacterial Species
Source: mBio. 2018 Nov 27;9(6):e01541-18. doi: 10.1128/mBio.01541-18 (PMC6282202; doi:10.1128/mBio.01541-18)
Supplement: TEXT S6 [file mbo006184194s6.pdf]

Mass Spectrometry

Tryptic peptides were analyzed with liquid chromatographic tandem mass spectrometry (LC-MS/MS). The LC-MS/MS system consisted of an LTQ XL IonTrap mass spectrometer (Thermo Scientific) equipped with a microflow ESI source and interfaced to an LCPackings Ultimate Plus Dual gradient pump, Switchos column switching device, and Famos Autosampler (Dionex). Two RP peptide traps C18 Pepmap 100 Dionex (0.30 mm x 5 mm) were used in parallel with two analytical BioBasic-C18 columns from Thermo Scientific (0.18 mm x 150 mm). Samples (6.5 μL) were injected and desalted on the peptide trap equilibrated with solvent A (3.5% acetonitrile v/v, 0.1% trifluoroacetic acid in water) at a flow rate of 30 μL/min. After valve switching, peptides were eluted in backflush mode from the trap onto the analytical column equilibrated in solvent B (5% acetonitrile v/v, 0.05% v/v formic acid in water) and separated using a 100-min gradient from 0 to 70% solvent C (80% acetonitrile v/v, 0.05% formic acid in water) at a flow rate of 1.5 μL/min.

Peak lists were generated using extract-msn (Thermo Fisher Scientific) within Proteome Discoverer 1.4.1. From raw files, MS/MS spectra were exported with the following settings: peptide mass range 350–5000 Da, minimal total ion intensity 500. The resulting peak lists were searched using SequestHT against a target-decoy *C. crescentus* protein database obtained from Uniprot (Proteome ID UP000001364). The following parameters were used: trypsin was selected with proteolytic cleavage only after arginine and lysine, number of internal cleavage sites was set to 1, mass tolerance for precursors and fragment ions was 1.0 Da, and considered dynamic modifications were +15.99 Da for oxidized methionine and +71.00 for acrylamide modified cysteine. Peptide matches were filtered using the q-value and Posterior Error Probability calculated by the Percolator algorithm ensuring an estimated false positive rate below 5%. The filtered Sequest HT output files for each peptide were grouped according to

the protein from which they were derived and their individual number of peptide spectral matches was taken as an indicator of protein abundance.
